# High pre- and postoperative symptom burden in non-responders to total knee arthroplasty

**DOI:** 10.1371/journal.pone.0233347

**Published:** 2020-05-28

**Authors:** Maren Falch Lindberg, Turid Undebakke Schweitz, Arild Aamodt, Caryl Gay, Anners Lerdal

**Affiliations:** 1 Department of Orthopaedic Surgery, Lovisenberg Diaconal Hospital, Oslo, Norway; 2 Department of Nursing Science, Faculty of Medicine, Institute of Health and Society, University of Oslo, Oslo, Norway; 3 Department of Family Health Care Nursing, University of California, San Francisco, California, United States of America; 4 Department of Research and Administration, Lovisenberg Diaconal Hospital, Oslo, Norway; 5 Department of Interdisciplinary Health Sciences, Faculty of Medicine, Institute of Health and Society, University of Oslo, Oslo, Norway; Cleveland Clinic, UNITED STATES

## Abstract

**Objectives:**

One in five patients does not improve in pain with walking (non-responders) 12 months after total knee arthroplasty (TKA). This longitudinal study investigated a broad range of symptoms before and after TKA and evaluated possible differences in symptom distress between responders and non-responders with regards to pain with walking after TKA.

**Methods:**

Prior to TKA surgery, 182 patients completed a demographic questionnaire and the Memorial Symptom Assessment Scale—Short Form (MSAS-SF). The MSAS-SF was repeated 12 months following TKA. Clinical data were extracted from medical records. Patients were categorized as responders or non-responders based on their trajectories of pain with walking assessed prior to surgery, on postoperative day 4, at 6 weeks, and at 3 and 12 months.

**Results:**

Overall, the most distressful preoperative symptoms were pain, lack of energy, difficulty sleeping, feeling drowsy, worrying, feeling bloated, and problems with sexual interest or activity. However, compared with patients classified as responders to TKA, non-responders had higher total symptom distress scores both preoperatively and 12 months postoperatively. Preoperatively, non-responders scored higher than responders on five of the seven most distressing symptoms (i.e., all except difficulty sleeping and feeling bloated), and 12 months postoperatively, non-responders scored higher than responders on six of the seven most distressing symptoms (i.e., all but feeling bloated). In a multivariate analysis, higher preoperative distress scores for pain and problems with sexual interest or activity were significant predictors of non-response to TKA, controlling for other relevant factors.

**Conclusions:**

Patients’ preoperative symptom burden may be a useful indicator of their risk for non-improvement following TKA surgery. Future studies need to evaluate the effect of reducing patients’ preoperative symptom burden on TKA outcomes.

## Introduction

Total knee arthroplasty (TKA) is a common procedure to reduce pain and improve functioning among patients with advanced osteoarthritis (OA) when non-surgical treatment no longer proves effective. Annually, the number of TKA procedures is steadily increasing with a total of 6905 primary knee replacements in Norway in 2017 [1] and an estimated 1,5 million procedures annually in the US [2]. While the procedure is highly successful for most patients, approximately 20% do not experience reduced pain with walking 12 months after TKA and continue to report pain [3]. In a meta-analysis that investigated a variety of studies on different factors and their relationship to persistent pain after TKA, symptoms and psychosocial characteristics were more frequently identified as risk factors, compared to demographic, perioperative or biomechanical factors [4]. The reasons why one in five patients experience a suboptimal result are still not fully understood, but appear to be multifactorial [5].

Symptomatology is an important part of the diagnostic process in knee OA. A symptom can be defined as "A subjective experience reflecting changes in the biopsychosocial functioning, sensation or cognition of an individual" [6] and can be divided into physical or psychological symptoms [7]. In knee OA, pain and stiffness are the cardinal symptoms [8], which in turn can interfere with knee functioning and lead to disability and poor quality of life. Patients with knee OA may also experience more general symptoms, and the presence and distress of certain symptoms may be risk factors for a poor outcome following TKA. For example, higher preoperative pain and multiple painful sites are considered risk factors for acute [9] and persistent pain following TKA [4]. Although patients with less preoperative pain have better postoperative outcomes, they also report less improvement than patients with higher preoperative pain levels [10].

While less recognized, fatigue and depression may also accompany OA and may occur in symptom clusters [11]. Psychological symptoms, such as anxiety and depression [12], may be particularly distressful as they have the capacity to affect a patient’s motivation, energy, coping strategies and adherence to rehabilitation, and may thereby interfere with postoperative pain, activity levels and functional outcomes [5]. For example, pain catastrophizing and poor mental health have been associated with persistent postoperative pain after TKA [4]. Previous studies have focused on investigating single symptoms, but this approach may be too simplistic. For example, the Ullensaker study [13], which assessed 10 musculoskeletal symptoms and 13 non-musculoskeletal symptoms in the general population in Norway, found that a substantial proportion of the population reported a variety of symptoms, and there was a strong relationship between the number of symptoms and functional status [13]. Moreover, the 23 symptoms they assessed explained almost half the variance in functional status, and the total number of symptoms could serve as a proxy for single symptoms [13]. Recent theories of symptom clusters suggest that multiple symptoms occurring together may cause a synergetic effect with greater negative impact on a person’s well-being than single symptoms [14]. In line with this theory, Jenkins and colleagues found a cluster of symptoms consisting of pain, fatigue and depression associated with poor function and quality of life in patients with OA [11]. Finally, results from the Ullensaker study [13] suggest that the traditional focus on only a few symptoms in OA patients may only capture the tip of an iceberg. As in the general population, patients on a waiting list for TKA may present with a multitude of other symptoms, not necessarily related to the OA itself. It is likely that symptoms that are unrelated to the OA condition will continue to be present after a TKA procedure and may continue to interfere with functional status and outcomes after TKA. Despite the potential negative impact of multiple symptoms on long-term outcomes after TKA, general symptomatology has so far received limited attention in patients undergoing TKA. To our knowledge, no studies have investigated a broad range of symptoms before and after TKA and evaluated their relationship to TKA outcomes.

To address this gap in knowledge and to achieve a better understanding of the factors that may be contributing to non-improvement following TKA, this study aims to investigate a broad list of symptoms that include somatic and psychological symptoms, which may or may not be related to the patients’ OA condition. The specific aims of this study are to describe preoperative symptoms in TKA patients and identify which symptoms are associated with non-response, defined as no improvement in pain with walking one year after surgery.

## Methods

This longitudinal study was carried out at Lovisenberg Diaconal Hospital in Oslo from October 2012 through September 2014. The Department of Orthopaedic Surgery is a high-volume clinic for elective orthopedic surgery, and patients are admitted from all regions of Norway, usually referred to the clinic by their general practitioner. The inclusion criteria were: diagnosis of OA, scheduled for primary TKA, age 18 years or older, and ability to read and write in Norwegian. Patients were excluded if they had a diagnosis of dementia. The study was approved by the Regional Medical Research Ethics Committee of Health South East of Norway (#2011/1755).

### Patients and procedures

All eligible patients received written information about the study either by mail prior to admission for TKA or in person on the day of admission. All participating patients signed an informed consent form prior to receiving the baseline questionnaire, usually on the day before surgery. Follow-up data on pain with walking was collected at 5 time points: prior to surgery, on postoperative day (POD) 4, at 6 weeks, and at 3 and 12 months following surgery. Follow-up data on symptoms was collected 12 months after surgery. All follow-up questionnaires were mailed to the participants with a pre-paid return envelope. Patients who did not respond received one reminder either by telephone or mail. Of the 245 patients invited to participate, surgery was cancelled for 6 patients and 33 declined to participate. From the 206 included patients, 2 were excluded after surgery due to postoperative confusion, 1 patient died following surgery, and 21 patients were excluded because of missing data on one or more of the symptom variables, leaving 182 patients for this analysis.

#### Patient management procedures

All patients followed standardized procedures for anesthesia, surgery and postoperative pain management and received a posterior cruciate-retaining implant with fixed modular bearing. The protocol for recovery was standardized. Patients were mobilized to standing and received physiotherapy with walking, flexion and extension of the knee starting on POD 1. They were allowed full weight bearing on the operated knee. Patients were usually discharged home on POD 3 or 4, and continued to receive weekly physiotherapy for up to 4–6 months.

### Measurements

Response to TKA was assessed using the Brief Pain Inventory (BPI). The BPI consists of four items that measure pain intensity rated on a scale from 0 (no pain) to 10 (pain as bad as you can imagine), seven items that measure pain interference with different domains of functioning rated on a scale from 0 (no interference) to 10 (interferes completely), and a body map to identify pain locations. For this study, pain interference with walking was used as the dependent variable. In a previous study from this study [15], using growth mixture modeling, two subgroups of patients were identified with distinct trajectories of pain interference with walking over time. The largest subgroup (i.e., responders) had a trajectory characterized by continuous improvement from before until 12 months after surgery. A smaller subgroup of approximately 22% of the patients (i.e., non-responders) had only temporary improvements up to 3 months following surgery, followed by a worsening of pain with walking, resulting in no improvement 12 months after surgery compared to preoperative pain levels.

General symptom experience was assessed using the Memorial Symptom Assessment Scale—Short Form (MSAS-SF) [16]. The distress dimension was selected because it provides the most important information in the MSAS [16]. Patients rated the occurrence and distress of 32 symptoms in the last 7 days on a 6-point scale ranging from 0 = Did not have the symptom, 0.8 = Not distressful, 1.6 = A little distressful, 2.4 = Somewhat distressful, 3.2 = Distressful, 4.0 = Very distressful. A total distress score was calculated as the mean score of all 32 symptoms, ranging from 0 to 4, with higher scores indicating more total symptom distress. The MSAS-SF has been validated in cancer patients [17] and has been used in medical and surgical patients [18, 19].

Preoperative demographic data were obtained by self-report and included age, sex, occupational status, and education level. Preoperative clinical data were extracted from patients’ medical records and included body mass index (BMI), number of comorbidities and American Society of Anesthesiologists (ASA) physical status classification [20].

### Statistical analysis

Data analysis was performed using the Statistical Package for Social Science version 24 (IBM, Armonk, NY). Growth mixture modeling (GMM) with full maximum likelihood estimation using Mplus version 7.3 [21] was performed to identify subgroups of responders and non-responders to surgery based on their trajectories of pain with walking from before until 12 months following surgery. This procedure has been described in detail elswhere [15].

Continuous data are reported as means and standard deviations, and categorical variables are reported as numbers and percentages. Wilcoxon signed rank tests were used to evaluate changes in the most distressful single symptoms (i.e., distress score >1), and total symptom burden before and 12 months after TKA, and Mann-Whitney U tests were performed to evaluate differences between responders and non-responders. A multivariate logistic regression model was performed with TKA response status (responder or non-responder) as the dependent variable, to identify which of the most distressful symptoms had the strongest relationship with non-response after TKA when controlling for other symptoms. Age and sex were included in the model as potential confounders. Effect sizes were calculated on differences in symptom scores between responders and non-responders, according to Cohen’s coefficient *d* (i.e., Small ≥ .2; Medium ≥ .5; Large ≥ .8) [22]. Level of statistical significance was set to p<0.05. Sample size calculation indicated that 180 patients (with a 3:1 allocation between groups) would be sufficient to detect a group difference of medium effect size (Cohen’s *d* of .5) with 80% power and an alpha level of .05.

## Results

Of the 182 patients included in this analysis, the majority were female, living with a partner and were not in paid work. The mean age was 68.6 years. The demographic and clinical characteristics of the sample are shown in Table 1.

**Table 1:** Demographic and symptom characteristics of patients (N = 182) prior to surgery.

Demographic characteristics			Mean	SD
Age (years)			68.6	9.1
			n	%
Sex—women			124	68.1
Married/partnered cohabitation status (n = 180)			109	60.6
Employment status Unemployed/retired (n = 180)			115	63.9
Education level College/university level (n = 177)			92	52.0
Preoperative clinical characteristics			Mean	SD
Body mass index			28.9	0.5
Number of comorbidities (0–5)			1.2	1.0
ASA physical status classification score			2.0	0.5
Symptom burden				
MSAS total symptom occurrence (i.e., number of symptoms)				
Prior to surgery			12.5	8.1
12 months after surgery			11.8	8.9
MSAS total distress score				
Prior to surgery			0.7	0.4
12 months after surgery			0.6	0.5
	Occurence		Distress	
Preoperative symptoms from the Memorial Symptom Assessment Scale	N	(%)	Mean	SD
Pain	168	92.3	2.4	1.0
Lack of energy	149	81.9	1.7	1.0
Difficulty sleeping	136	74.7	1.6	1.2
Feeling drowsy	132	72.5	1.5	1.1
Worrying	130	71.4	1.4	1.1
Feeling sad	98	53.8	0.9	1.0
Feeling irritable	95	52.2	0.8	0.9
Feeling bloated	90	49.5	1.0	1.1
Problems with sexual interest or activity	86	47.3	1.0	1.3
Feeling nervous	83	45.6	0.7	1.0
Swelling of arms or legs	79	43.4	0.8	1.0
Difficulty concentrating	79	43.4	0.8	1.0
Dry mouth	76	41.8	0.8	1.1
Sweats	73	40.1	0.7	1.0
Numbness/tingling in hands/feet	68	37.4	0.6	0.9
Shortness of breath	59	32.4	0.6	0.9
Dizziness	57	31.3	0.4	0.7
Cough	54	29.7	0.4	0.7
Problems with urination	52	28.6	0.4	0.8
Itching	52	28.6	0.4	0.8
Lack of appetite	49	26.9	0.3	0.7
Constipation	48	26.4	0.4	0.8
“I don’t look like myself”	45	24.7	0.4	0.7
Diarrhea	44	24.2	0.3	0.6
Changes in skin	43	23.6	0.3	0.7
Nausea	39	21.4	0.3	0.6
Mouth sores	36	19.8	0.2	0.6
Hair loss	34	18.7	0.3	0.7
Difficulty swallowing	33	18.1	0.2	0.4
Weight loss	31	17.0	0.2	0.5
Change in the way food tastes	27	14.8	0.2	0.5
Vomiting	25	13.7	0.1	0.3

ASA = American Society of Anesthesiologists

### Symptom distress before and after surgery

**Fig 1:**
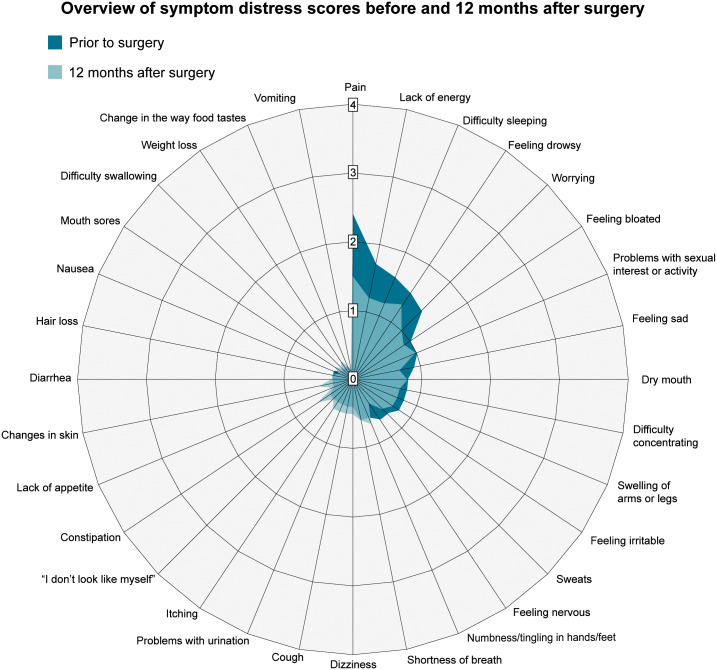
Overview of symptom distress scores before and 12 months after surgery. Symptom distress scores from the MSAS, higher scores indicate higher symptom distress.

Of the 32 symptoms, the 7 most prevalent before surgery (i.e., reported by more than 50% of the sample to have occurred in the prior 7 days) were: pain, lack of energy, difficulty sleeping, feeling drowsy, worrying, feeling sad, feeling irritable (Table 1). In addition, the 7 most distressful preoperative symptoms were: pain, lack of energy, difficulty sleeping, feeling drowsy, worrying, feeling bloated, and problems with sexual interest or activity (Table 1). The pattern of distress scores for the 32 symptoms before and 12 months after surgery is illustrated in Fig 1. Patients’ total distress scores were significantly lower (p = 0.001) at 12 months (mean 0.6, SD 0.5) compared to before surgery (mean 0.7, SD 0.4). Given that the effect size of this difference is small (Cohen’s d = 0.2), there is likely minimal clinical significance of this decline in total distress scores.

### Preoperative symptom distress in responders and non-responders

Patients classified as non-responders had significantly higher preoperative total symptom distress scores (mean 0.9, SD 0.4) than patients classified as responders (mean 0.6, SD 0.4; p = 0.001), and the effect size for the difference was moderate to large (Cohen’s d .75). Mann-Whitney U tests with each of the 7 most distressful symptoms showed that the non-responders also had higher distress scores than the responders on the following five single preoperative symptoms: pain, lack of energy, feeling drowsy, worrying, and problems with sexual interest or activity (Table 2).

**Table 2:** Differences in symptom distress between responders and non-responders before and 12 months after TKA.

	Responders (n = 140, 77%)		Non-responders (n = 42, 23%)			
*Preoperative symptoms*	Mean	SD	Mean	SD	p-value*	Cohen’s *d*
Pain	2.3	1.0	2.8	0.8	.001	0.6
Lack of energy	1.6	1.0	2.1	0.9	.001	0.5
Difficulty sleeping	1.6	1.3	1.8	1.2	.300	0.2
Feeling drowsy	1.3	1.1	2.0	1.2	.002	0.6
Worrying	1.2	1.0	1.8	1.2	.006	0.5
Feeling bloated	0.9	1.1	1.0	1.0	.714	0.1
Problems with sexual interest or activity	0.9	1.2	1.5	1.4	.003	0.5
Total symptom distress**	0.6	0.4	0.9	0.4	.001	0.8
*Symptoms at 12-month follow-up*	Mean	SD	Mean	SD	p-value*	
Pain	1.2	0.9	2.3	1.0	< .001	1.2
Lack of energy	1.1	1.0	1.7	1.1	.001	0.6
Difficulty sleeping	1.1	1.1	1.7	1.3	.004	0.5
Feeling drowsy	1.1	1.0	1.9	1.0	< .001	0.8
Worrying	0.8	0.9	1.4	1.1	.001	0.6
Feeling bloated	0.8	1.0	1.2	1.3	.181	0.3
Problems with sexual interest or activity	0.8	1.1	1.5	1.5	.011	0.5
Total symptom distress**	0.5	0.4	0.9	0.6	< .001	0.8

Cohen’s *d* effect sizes: 0.2 = small, 0.5 = moderate, 0.8 = large

Abbreviations:

*Mann-Whitney U tests

**Calculated from the 32 symptoms of the Memorial Symptom Assessment Scale

### Symptom distress 12 months after surgery in responders and non-responders

Patients classified as non-responders also had significantly higher total symptom distress scores 12 months after surgery (mean 0.9, SD 0.6), compared to patients classified as responders (mean 0.5, SD 0.4; p<0.001)).

Mann-Whitney U tests with each of the 7 most distressful symptoms showed that non-responders had higher distress scores than responders on the following six single postoperative symptoms: pain, lack of energy, difficulty sleeping, feeling drowsy, worrying, and problems with sexual interest or activity (Table 2).

### Changes in symptom burden over time among responders and non-responders

Responders had significant improvements in their total distress scores from before surgery (mean 0.6, SD 0.4) to 12 months after surgery (mean 0.5, SD 0.4; p<0.001), and the effect size for the difference was moderate (Cohen’s *d* = 0.5). Non-responders had no improvement in total distress scores 12 months after surgery (mean 0.9, SD 0.6), compared to their preoperative scores (mean 0.9, SD 0.4; p = 0.70).

Responders had significant improvements over time in the following 4 symptoms: pain (p<0.001), lack of energy p<0.001), difficulty sleeping (p<0.001), and feeling drowsy (p = 0.03). Non-responders had significant improvements over time in only the following 2 symptoms: pain (p = 0.006) and lack of energy (p = 0.007).

### Preoperative symptoms as predictors of non-response after TKA

As shown in Table 3, the 7 most distressful preoperative symptoms (i.e., pain, lack of energy, difficulty sleeping, feeling drowsy, worrying, feeling bloated, problems with sexual interest or activity) were entered into a multivariate logistic regression model, with TKA response status as the dependent variable. Higher distress scores for pain (OR 1.8, p = 0.04) and problems with sexual interest or activity (OR 1.4, p = 0.04) were significant predictors of non-response to TKA, after controlling for all other factors in the model.

**Table 3:** Preoperative symptom predictors of non-response 12 months after TKA.

	Wald	OR	95% CI		P-value
*Demographic variables*					
Age (years)	1.5	1.0	1.0	1.1	.23
Male sex (female as reference)	0.4	1.3	0.3	1.8	.52
Symptoms*					
Pain	4.4	1.8	1.04	3.1	.04
Lack of energy	2.0	1.5	0.9	2.7	.15
Feeling drowsy	2.5	1.5	0.92	2.4	.11
Difficulty sleeping	2.9	0.7	0.5	1.1	.09
Feeling bloated	3.5	0.7	0.5	1.0	.06
Worrying	2.1	1.4	0.9	2.1	.15
Problems with sexual interest or activity	4.0	1.4	1.01	1.8	.04

Model fit parameters: Omnibus test: p<0.001. Hosmer and Lemeshow test: Chi-square = 8.97, df = 8, p = 0.35.

Cox & Snell R-square: 15.5%, Nagelkerke R-square: 23.5%. -2 Log likelihood: 165.9.

P < .05 is considered statistically significant.

Dependent variable: Responders (coded 0) and non-responders (coded 1) after TKA.

Abbreviations: CI = Confidence Interval. OR = Odds Ratio

*7 most distressful symptoms from the Memorial Symptom Assessment Scale—Short Form

## Discussion

This study is the first to evaluate a broad list of 32 general symptoms before and after TKA, as well as their association with outcomes after TKA. Prior studies on TKA have focused primarily on symptoms directly related to OA, yet our findings revealed that OA patients experience a broad range of general symptoms prior to surgery, several of which seem unrelated to their knee OA. While the majority of patients experienced significant reductions in symptom burden during the 12 months following surgery, a subgroup of patients, previously classified as non-responders, had significantly less improvement than those classified as responders. In addition, the non-responders had a significantly higher total preoperative symptom burden than responders, as well as higher scores on five pre- and postoperative symptoms (i.e., pain, lack of energy, feeling drowsy, worrying, problems with sexual interest or activity). Of the 32 symptoms, pain and problems with sexual interest or activity stood out as the main predictors of non-improvement after TKA.

Not surprisingly, higher preoperative pain distress was the most important predictor of non-improvement following TKA, with a large effect size (Cohen’s *d* = 1.2). Our results are in line with a variety of studies showing that a high level of preoperative pain is a known risk factor for non-improvement and chronic pain following TKA [4]. Clinicians should therefore address this issue with patients prior to surgery. Patients who report high levels of preoperative distress due to pain may benefit from prehabilitation [23] combined with an individualized pain management plan and rehabilitation following surgery to reduce their risk of persistent postoperative pain and improve their outcomes.

Among the 32 symptoms assessed, problems with sexual interest or activity stood out as the second most important predictor of non-improvement. Of note, in analyses of which activities were most important to patients following TKA, return to sexual activity have ranked higher than any other activities [24, 25]. These findings were not supported in a study of female Asian TKA patients [26] or in British patients undergoing either TKA or total hip replacement (THA). This may be due to cultural, gender- and age-related differences in these samples. While this topic is not well studied in TKA populations, a systematic review of patients undergoing THA [27] concluded that sexual activity is important to patients, yet rarely addressed by surgeons. Our findings suggest that in addition to being a desired outcome, a healthy sexual life before surgery may also be a protective factor associated with better functional results after TKA. As pointed out by a systematic review [28], TKA patients can expect to return to sexual activity an average of 2.4 months following surgery, and improvements in sexual activity are likely to be greater in younger patients and to increase over time. As this is the first study to investigate the relationship between preoperative sexual problems and TKA outcomes, our results point to the need for future studies to investigate this topic more in-depth and with more specific measurements.

Non-responders also experienced higher preoperative levels of distress related to lack of energy, feeling drowsy, and worrying, and these symptoms were more likely to persist in non-responders than in responders. These factors could be addressed and modified prior to surgery. Excessive worrying is among the most common symptoms of anxiety and depression [29]. Similar to our findings, several studies have shown correlations between preoperative psychological distress [30], depression [31] and surgical outcomes after TKA, and there is growing evidence suggesting that pain catastrophizing is among the strongest predictors for a poor outcome following TKA [4]. Pain catastrophizing is characterized by rumination and worrying about pain [32], and has been linked to a fear of movement in patients with OA [33], which in turn may interfere with activity following TKA [34]. In a recent quasi-experimental trial in patients with high pain catastrophizing scores, preoperative pain coping training resulted in less pain and better functional outcomes following TKA [35]. Future studies should investigate the effectiveness of such interventions in randomized controlled trials.

A lack of energy is a common description of the feeling of fatigue [36], and combined with the feeling of drowsiness, these symptoms may be indicative of increased fatigue levels [37]. Despite 35% of patients with OA experiencing severe fatigue [36], few studies have investigated this symptom and its impacts on outcomes following TKA surgery. In a qualitative study, OA patients reported high and disabling fatigue levels, described as a type of exhaustion and tiredness, and enhanced by OA pain and poor sleep [38]. In a recent systematic review, it is suggested that the relationship between poor sleep and increased fatigue is mediated by joint pain [39]. Furthermore, fatigue has been found to be a predictor of lower activity levels in OA patients [40]. Because the underlying mechanisms of OA-related fatigue are not clear or linked to one specific underlying condition, but rather are described as a multidimensional phenomenon, it is often considered similar to generalized fatigue [39], and thus, few interventions are available to modify it. However, individualized plans for bouts of activity and rest are recommended, and moderate aerobic exercise programs have shown promise, with a reduction in fatigue that lasted for up to 3 months [39]. Interestingly, we found that patients classified as non-responders had higher pre- and postoperative fatigue levels than the responders. These findings indicate that patients who do not respond to TKA may represent a different phenotype than the responders, suggesting that patients may have different underlying mechanisms that sustain their fatigue. Future studies of the underlying mechanisms of fatigue should use a multidimensional approach [39] in order to yield findings that can be used to develop interventions to reduce fatigue in this patient group.

The average distress scores for the 32 symptoms were relatively low. However, a subset of patients defined as non-responders reported significantly higher total symptom distress, as well as higher distress scores for seven symptoms, compared to responders. For example, patients in this sample reported experiencing an average of 12 symptoms in the past week, which is 50% higher than the number of symptoms reported in a sample of community-dwelling elderly patients who had been hospitalized >3 times during the last year [41]. In addition, this sample’s distress scores for pain, lack of energy, difficulty sleeping, and feeling drowsy were even higher than those reported in a sample of patients awaiting treatment for colorectal cancer [42]. These finding suggest that OA patients experience a variety of symptoms to varying degrees, which may or may not be related to their OA. Our findings are in line with the results of a study investigating the occurrence of 23 symptoms in the Norwegian general population, which found that a substantial proportion of the participants reported a variety of symptoms [13]. In that study, the total burden of symptoms explained almost half of the variation in patients’ functional ability, suggesting a synergistic effect of the total number of symptoms instead of the specific effects of single symptoms [13]. Traditionally, there has been little focus on preoperative symptoms that are unrelated to knee OA and their associations with outcomes after TKA surgery. Despite being unrelated to OA, such symptoms may have a significant, perhaps even stronger impact on outcomes following TKA. For example, symptoms that are related to the OA itself, or related to the pain associated with OA, are likely to gradually diminish after surgery [43]. In contrast, symptoms that are unrelated to the patient’s OA condition are most likely related to other factors and are therefore more likely to persist after surgery, potentially interfere with rehabilitation, and thereby have potentially negative impacts on TKA outcomes.

When no explanations for symptoms are found, they are often referred to as medically unexplained symptoms. Medically unexplained symptoms (MUS) can be defined as persistent bodily complaints for which adequate examination does not reveal sufficiently explanatory structural or other specified pathology [44]. Irritable bowel syndrome (IBS) and fibromyalgia are conditions characterized by medically unexplained symptoms, of which several are similar to symptoms identified in our study. For example, fibromyalgia is associated with widespread pain, fatigue, cognitive dysfunction, and sleep disruption [45], and abdominal pain and bloating are among the most common features of IBS [46]. While these conditions are still not well understood, they have been linked to central mechanisms and inflammation [46, 47]. For example, the presence of fibromyalgia was a robust predictor of a poor outcome following TKA [48], even among patients who scored in the higher range but below the fibromyalgia phenotype cutoff. In a sample of TKA patients with fibromyalgia symptoms [49], 32% of the patients experienced only temporary improvements in their fibromyalgia symptoms that lasted up to one month, followed by a relapse in symptoms over the next five months, suggesting involvement from central mechanisms that may have sustained their symptoms. While patients in our sample reported several of the characteristic symptoms involved in fibromyalgia, we do not have information on whether patients in our sample had this diagnosis. Our findings were adjusted for comorbidities in general.

Our study had several limitations. First, patients were recruited from a single surgical department, which may have limited the generalizability of the results. However, we aimed to overcome this limitation by including patients from all parts of Norway. More than half of the sample lived in the Oslo region or nearby. Second, our sample had a slightly lower BMI compared to patients in TKA populations from the USA [50, 51] and Ireland [52], which likely reflects differences in BMI for the general population across these countries [53]. Nonetheless, these BMI differences may limit the generalizability of our study findings to populations with similar BMI profiles. Third, we do not have data on previous injuries that may have led to the OA condition and could therefore not evaluate the impact of different OA etiologies on the study findings. Finally, the measures used in this study are all self-report, as is standard for most symptom assessments, but may have been influenced by other factors, such as optimism or catastrophizing. As the impact of such factors on self-reported symptom measures has not been adequately evaluated, the role of response bias on this study’s findings is unknown, and future studies in this area are needed. Despite these limitations, this study had a number of key strengths, including the prospective design, the use of validated instruments and the low attrition rate.

## Conclusions

Compared to patients who improved after TKA, non-responders had higher preoperative symptom distress scores, both overall and for the symptoms of pain, lack of energy, feeling drowsy, worrying, and problems with sexual interest or activity. These findings suggest the need to develop a screening tool that includes a wide range of symptoms, which could assist clinicians in identifying patients at high risk of a poorer outcome if they decide to undergo TKA. These findings also indicate that clinicians need to be aware of patients who present with multiple symptoms and high levels of pain prior to TKA, as they may be at higher risk for non-improvement following surgery. Our findings may have implications for TKA patient selection. Furthermore, future studies should develop and test preoperative interventions to reduce symptom burden, on outcomes following TKA.
